# Corrigendum

**DOI:** 10.1002/prp2.429

**Published:** 2018-09-17

The original version of this paper, published in *Pharmacology Research & Perspectives* Journal, 2014, 2(6), (DOI: 10.1002/prp2.70), contains an error in the fifth author's name

The correct author listing is:

Amartya Mishra, Jayaraman Vinayagam, Sourav Saha, Sayan Chowdhury, Somenath **Roy Chowdhury**, Parasuraman Jaisankar & Hemanta K. Majumder
